# Phylogenetic and comparative analyses of *Hydnora abyssinica* plastomes provide evidence for hidden diversity within Hydnoraceae

**DOI:** 10.1186/s12862-023-02142-w

**Published:** 2023-07-18

**Authors:** Elijah Mbandi Mkala, Matthias Jost, Xiang Dong, Geoffrey Mwachala, Paul Mutuku Musili, Stefan Wanke, Guang-Wan Hu, Qing-Feng Wang

**Affiliations:** 1 CAS Key Laboratory of Plant Germplasm and Specialty Agriculture, Wuhan Botanical Garden, Chinese Academy of Sciences, Wuhan, CN-430074 China; 2 Sino-Africa Joint Research Center, Chinese Academy of Sciences, Wuhan, CN-430074 China; 3 University of Chinese Academy of Sciences, Beijing, CN-100049 China; 4 Institut für Botanik, Technische Universität Dresden, 01062 Dresden, Germany; 5 East African Herbarium, National Museums of Kenya, P. O. Box 451660-0100, Nairobi, Kenya; 6 Departamento de Botánica, Instituto de Biología, Universidad Nacional Autónoma de México, Mexico City, Mexico

**Keywords:** Heterotrophy, Kenya, Taita hills, Plastome condensation, Monophyly, Piperales

## Abstract

**Background:**

To date, plastid genomes have been published for all but two holoparasitic angiosperm families. However, only a single or a few plastomes represent most of these families. Of the approximately 40 genera of holoparasitic angiosperms, a complete plastid genome sequence is available for only about half. In addition, less than 15 species are currently represented with more than one published plastid genome, most of which belong to the Orobanchaceae. Therefore, a significant portion of the holoparasitic plant plastome diversity remains unexplored. This limited information could hinder potential evolutionary pattern recognition as well as the exploration of inter- and intra-species plastid genome diversity in the most extreme holoparasitic angiosperms.

**Results:**

Here, we report the first plastomes of Kenyan *Hydnora abyssinica* accessions. The plastomes have a typical quadripartite structure and encode 24 unique genes. Phylogenetic tree reconstruction recovers the Kenyan accessions as monophyletic and together in a clade with the Namibian *H. abyssinica* accession and the recently published *H. arabica* from Oman. *Hydnora abyssinica* as a whole however is recovered as non-monophyletic, with *H. arabica* nested within. This result is supported by distinct structural plastome synapomorphies as well as pairwise distance estimates that reveal hidden diversity within the *Hydnora* species in Africa.

**Conclusion:**

We propose to increase efforts to sample widespread holoparasitic species for their plastid genomes, as is the case with *H. abyssinica*, which is widely distributed in Africa. Morphological reinvestigation and further molecular data are needed to fully investigate the diversity of *H. abyssinica* along the entire range of distribution, as well as the diversity of currently synonymized taxa.

**Supplementary Information:**

The online version contains supplementary material available at 10.1186/s12862-023-02142-w.

## Introduction

Holoparasitism has evolved in ten out of twelve parasitic angiosperm lineages, resulting in a great diversity of genera and species [1]. The trophic change from auto- to full heterotrophy is accompanied by drastic changes to the plastid genome [2], culminating in highly condensed plastomes with respect to gene content as well as genome size [3–5]. Alongside the gene function losses, changes to the otherwise extremely conserved plastome structure are often observed. Many so called minimal plastomes do not maintain the typical quadripartite structure due to the loss of one of the inverted repeat copies [3–7]. Additionally, many smaller inversions and translocations can be observed [2, 8], which may be correlated to the increased AT content of plastomes of heterotrophic plants [9, 10]. Based on published data, general models were proposed to explain lineage-independent patterns of plastome condensation during adaptation to a heterotrophic lifestyle [7, 9–12]. However to date, no such models exist for the “final stages of plastome condensation”, the minimal plastomes, as a variety of different structures and gene contents have been observed [3–7]. This could either be due to lineage-specific factors being at play or be at least partially, due to lack of data.

At the time of this study, all holoparasitic angiosperm families are represented with at least a single published plastome, with the exception of Mystropetalaceae (Santalales) and Rafflesiaceae (Malpighiales). For the latter, independent studies have hypothesized the complete plastid genome loss [13, 14]. The NCBI database reviewing (https://www.ncbi.nlm.nih.gov/) reveals that, out of the ~ 40 genera of holoparasitic angiosperms, only about half of them have full plastid genome sequences available. In addition, less than 15 species are currently represented by more than one published plastid genome (mostly limited to the Orobanchaceae). Therefore, a significant fraction of the diversity remains currently unexplored. This limited information might hinder, among others, potential evolutionary pattern recognition as well as explorations on inter-species and intra-species diversity of plastid genomes.

Plastomes of Hydnoraceae are highly condensed with respect to genome size and gene content and among the smallest known to date [7, 8]. The typical quadripartite plastome structure is shown missing in genus *Prosopanche* de Bary as well as in *Hydnora esculenta* Jum. & H. Perrier [6, 8]. The remaining published species of *Hydnora* Thunb., clustered in two clades, show differences with respect to their IR positioning and content, allowing for multiple evolutionary scenarios of IR and direct repeat evolution [8]. Apart from structural differences, the plastomes show an extreme degree of divergence on nucleotide sequence level [6]. The nucleotide composition is highly biased towards A and T, with an average of 23.1% GC in genus *Hydnora* and average of 21.7% GC in *Prosopanche* [8], compared to an average of 34–39% in photosynthetic angiosperms [15].

The family Hydnoraceae consists of the two genera *Hydnora* [16] and *Prosopanche* [17]. The former being distributed from Southern Africa to the Arabian Peninsula and Madagascar, the latter from South to Central America [18, 19]. This holoparasitic family of root parasites is among the oldest parasitic angiosperm lineages [20], and places within the magnoliid order Piperales. Together with Aristolochiaceae, Asaraceae, and Lactoridaceae they form the perianth-bearing clade within the order [21]. *Prosopanche* consists of seven species [22] whereas eight species are currently recognized in *Hydnora* [23, 24]. Hydnoraceae are unique among angiosperms due to their extreme loss of morphological features, e.g. the loss of leaves or scales and the ability to release a rotting smell that attracts beetles for pollination, hence described as the “strangest plants on earth” [25]. The flowers and fruits are the only aboveground parts of the mostly subterranean Hydnoraceae plants. The only exception to this is *H. triceps* Drege & E. Mey., which is known to complete its entire life cycle below ground [26, 27]. According to the Flora of tropical East Africa (FTEA), three *Hydnora* species have been recorded in Kenya, namely *H. sinandevu* Beentje & Q. Luke, *H. abyssinica* A. Braun, and *H. africana* Thunb [28]. These species are commonly being used as medicine, as source of food, and their roots are essential for tanning leather [24]. *Hydnora abyssinca* is described to have the largest distribution range among the members of the family, ranging from Southern Africa to northern East Africa [29]. Based on a comprehensive analysis of herbarium specimens, Musselman [18] recognized *H. abyssinica*, and treated *Hydnora johannis* Becc. and *Hydnora solmsiana* Dinter as synonyms of the first [18]. Additionally, *Hydnora* species are rarely found and collected due to their cryptic nature and seasonal appearance. Herbarium material preservation is often poor, which has made systematic revisions challenging. Apart from what is known about the natural history of *Hydnora* [18, 23, 26, 30]; there is only limited knowledge available on the morphogenesis and growth of this strange group of parasitic plants.

Here, we explore inter-species plastome diversity in Hydnoraceae for the first time. We report seven new plastomes of *Hydnora abyssinica* from Kenya and compare them with published plastomes of *H. abyssinica* from Namibia as well as to *H. arabica* Bolin & Musselman from Oman. Based on our genome reconstruction and a resolved phylogenetic hypothesis including a wider sampling in Hydnoraceae, we analyze their structure, nucleotide composition, as well as codon usage biasness.

## Results

### The plastomes of Kenyan *Hydnora abyssinica*

We reconstructed seven circular plastid genomes for the newly sequenced *H. abyssinica* accessions (H0 - H6). The plastomes are identical with respect to gene content and quadripartite plastome structure, with little variation on sequence level. The average plastome size is 24,549 bp, with accession H2 (24,526 bp) having the smallest and accession H4 (24,565 bp) having the largest plastome size/length(Additional file 1: Table S1). The majority of the length difference for H2 stems from shorter IR copies, whereas the size increase for H4 stems from a slightly increased large single copy region. The plastomes show little variation with respect to overall nucleotide composition bias, with a 23.2% of GC in all accessions, except H3 where we recorded 23.5% of GC instead (Additional file 1: Table S1). We included ambiguous characters at plastome positions with low readmapping support and within the *ycf*2 gene of accessions H3 and H5 to allow for circularization. PCR and Sanger sequencing were used to verify the regions, for example, verify the IR – SSC ssc region.

**Fig. 1:**
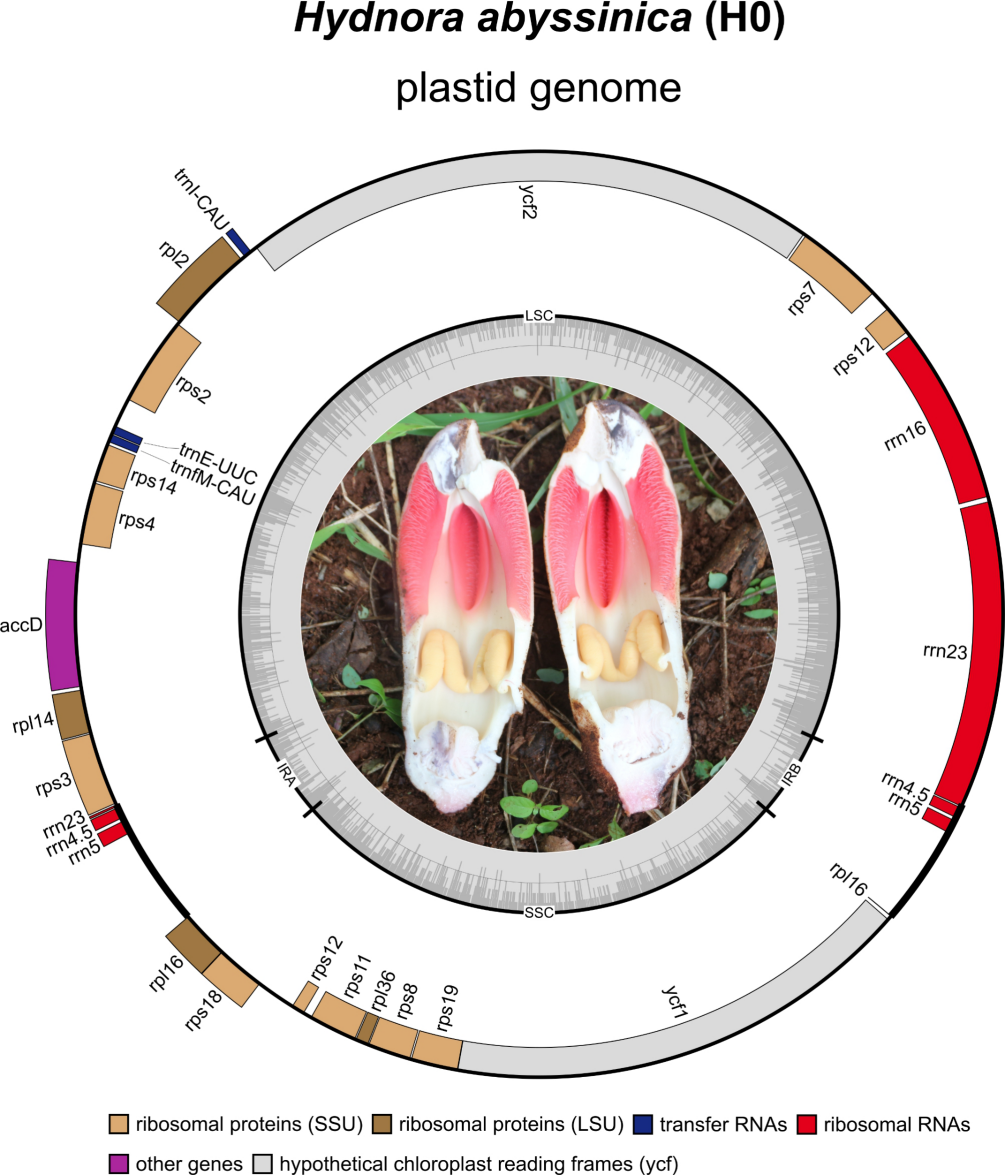
The plastid genome of Kenyan *Hydnora abyssinica* and its flower. The accession H0 is depicted as representative of accessions H0 – H6. Genes inside the outer circle are transcribed clockwise, genes on the outside are transcribed counter clockwise. The inner grey circle visualizes the GC content across the plastome. The center of the figure contains a picture of a longitudinally dissected *Hydnora abyssinica* flower from Kenya shortly before anthesis

The plastid genomes of all seven Kenyan *H. abyssinica* accessions contain an identical gene set, consisting of 24 unique genes (Fig. 1). The gene set is comprised of 17 protein-coding genes, four rRNA genes as well as three tRNAs. The single remaining intron is the *trans*-spliced intron of the *rps*12 gene (Fig. 1). Two complete (*rrn*4.5, *rrn*5) and two partial (*rrn*23, *rpl*16) genes are duplicated in the inverted repeats of the Kenyan *H. abyssinica* accessions (Fig. 1).

### Phylogenetic placement of Kenyan *Hydnora abyssinica*

**Fig. 2:**
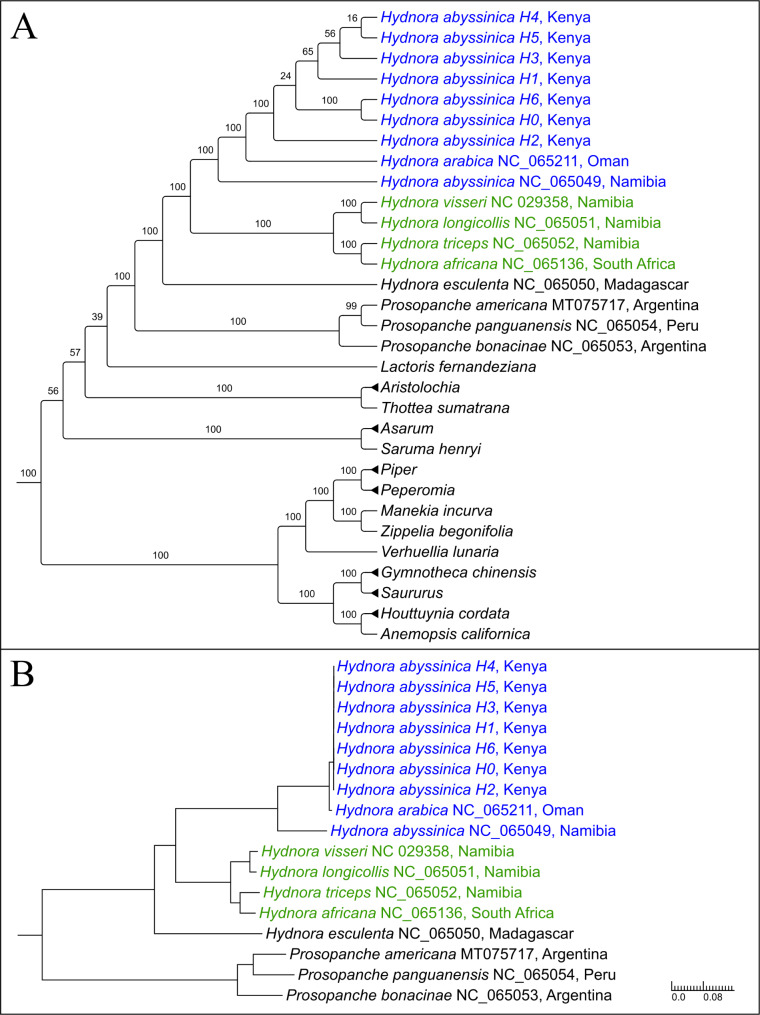
Phylogenetic placement of Kenyan *Hydnora abyssinica* accessions based on an 83 plastid-loci data set as clado- (**A**) and phylogram (**B**). Note that *H.abyssinica*, with H. arabica nested within, is not recovered as monophyletic, with *Hydnora arabica.* Outgroups are represented in a condensed form in (**A**) and completely removed in (**B**) to allow for better focus on the study group. The complete trees can be found in Figure S1. Inferences were calculated using RAxML. Bootstrap support values are shown along the respective nodes. Members of Hydnora clades I and II (based on Jost et al. [8] are highlighted in green and blue, respectively

Phylogenetic tree reconstruction of an 83-plastid loci data set recovers the Kenyan *H. abyssinica* accessions as monophyletic with 100 bootstrap support (BS). An equally high BS is recovered for the sister group relationship of *H. abyssinica* accessions H0 and H6. However, other nodes among the Kenyan accessions receive low to no BS (Fig. 2A, Fig. S1). The newly sequenced accessions place sister to *H. arabica* with 100 BS and successively sister to the Namibian *H. abyssinica* (BS 100). *Hydnora* formally referred to as Hydnora clade II was expanded by Jost et al. [8],using the newly sequenced plastomes.

Branch lengths within Hydnora clade II are relatively short, with the longest branches separating the Namibian *H. abyssinica* from *H. arabica* and the Kenyan sampling (Fig. 2B). Within the latter, we observed short branch lengths stemming from low sequence variation compared to what is seen in Hydnoraceae in general (Additional file 2: Table S2).

### Structural plastome comparisons among *H. abyssinica* and relatives

**Fig. 3:**
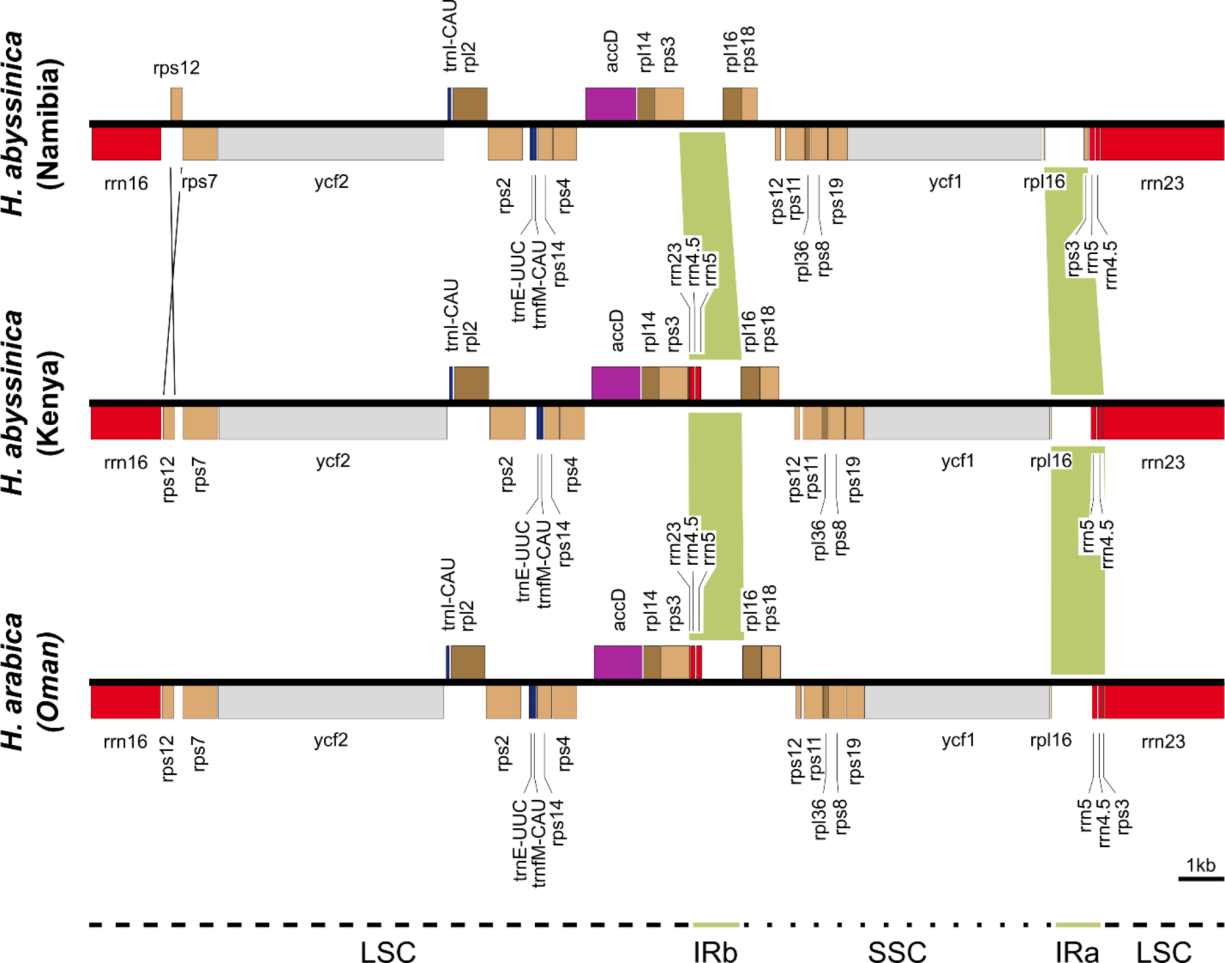
Structural plastome comparision among Namibian and Kenyan *H. abyssinica* and *H. arabica* (Hydnora clade II). The linear plastomes highlight incongruences and similarities among Namibian and Kenyan *H. abyssinica* and *H. arabica*. The green area highlights the positions and size of the respective IRs. Plastome regions (LSC, SSC, and IR copies) are indicated below

Although branch lengths among the focal taxa are relatively short, a structural plastome comparison reveals striking differences (Fig. 3). The orientation of the *rps*12 exon 2 is the only exception to an otherwise congruent gene order and orientation. The exon is inverted in Namibian *H. abyssinica* compared to the Kenyan accessions and *H. arabica* (Fig. 3). Additional differences concern the gene content of the Hydnora clade II IRs, in the plastome of Namibian *H. abyssinica*, the repeat consists of partial copies of *rpl*16 and *rps*3, whereas the IR of Kenyan *H. abyssinica* accessions contains full-length copies of *rrn*4.5 and *rrn*5, along with partial copies of *rrn*23 and *rpl*16 (highlighted green in Fig. 3). The IRs of *H. arabica* are identical to the ones of Kenyan *H. abyssinica* with respect to gene content, while additionally including a partial copy of *rps*3 (Fig. 3). Lastly, the *rps*19 and *ycf*1 genes do not partially overlap in the plastome of Namibian *H. abyssinica* and instead have a four bp spacer in between, whereas the genes show a four bp overlap in *H. arabica* and Kenyan *H. abyssinica*.

### Relative synonymous codon usage

RSCU analysis of the Hydnoraceae protein-coding genes reveals an extreme bias towards A and T nucleotides (Additional file 3: Table S3), complementary to the plastomes’ overall bias (Additional file 1: Table S1). For all amino acids, the most used codons correspond to the ones with the highest AT content (Additional file 3: Table S3). The preferred codons are mostly consistent among the sampled *Hydnora* and *Prosopanche* species, but also between the genera and the outgroup (*Aristolochia*, *Lactoris*, and *Thottea*). For four amino acids however, namely alanine, glycine, isoleucine, and serine, differences within the parasitic family have been uncovered (Additional file 4: Table S3). For alanine, the most used codon in *Prosopanche* and Hydnora clade I (*H. africana*, *H. longicollis* Welw, *H. triceps*, and *H. visseri* Bolin, E. Mass & Musselman) was GCT. In Hydnora clade II and *H. esculenta* however, GCA was the preferred codon, with GCT being only the second most used synonymous codon. The same pattern has been identified for the preferred codons for isoleucine. *Prosopanche* and Hydnora clade I favor ATA, whereas Hydnora clade II and *H. esculenta* favor ATT. Here, *P. bonacinae* Spe*g.* is considered an outlier, also with ATT estimated to be the most used codon (Additional file 3: Table S3). The predominant use of synonymous codons for serine highlights a case of different codon usage between *Prosopanche* and *Hydnora*. The most used synonymous serine codon for the latter is TCA, whereas *Prosopanche* favors TCT, with equal proportional use of TCT and AGT in *P. bonacinae* and *P. americana*. The most diverse use of preferred synonymous codons was observed for glycine, where Hydnoraceae species shifted between GGA and GGT (Additional file 3: Table S3). Hydnora clade II, *H. visseri* and *P. americana* prefer GGA, whereas the remainder of the sampling favor GGT. However, *H. longicollis* is estimated to have equal usage of GGA and GGT.

Comparison of the RSCU among Hydnoraceae and heterotrophic outgroup taxa reveals a much more even usage of synonymous codons for the latter, with only a low fraction of codons showing ≤ 10% usage (Additional file 3: Table S3).

## Discussion

### Hidden diversity within *Hydnora abyssinica* and relatives

The non-monophyly of *H. abyssinica* accessions from Kenya and Namibia, supported by structural plastome evidence, serves as first phylogenomic evidence for hidden diversity within *H. abyssinica*. The observed plastome reconfiguration, along with the inverted repeat content, compliment a supported tree reconstruction (Figs. 2 and 3). Based on these lines of evidence and to aid in clarification for future studies, we propose to recognize the Kenyan and Namibian accessions of *H. abyssinica* as distinctly different species. This proposal receives support by a recent morphological systematic revision, reconsidering *H. arabica* from Oman as *H. hanningtonii* and *H. abyssinica* from Namibia as *H. solmsiana* [31]. Following this taxonomic revision, our study provides the first plastid genomes for *Hydnora abyssinica*. However, it needs to be noted that the current molecular evidence for splitting species stems from plastid data only. Further nuclear data will be needed to confirm our findings as well as the new systematic treatments in general.

Morphological characterization of many holoparasites, including Hydnoraceae, proves difficult due to a lack of features combined with the uncertain assignment of some remaining ones [31]. Furthermore, herbarium specimens are often poorly preserved and mostly floral structures are documented, whereas conserved underground organs are rare. *Hydnora abyssinica* shows the widest distribution range among all members of Hydnoraceae. It is occurring in arid and semi-arid biomes from South Africa to northern East Africa [23–25, 27, 28, 31–34]. An approximately equally broad distribution range was proposed for closely related *Prosopanche americana* in the New World. Meanwhile, several additional *Prosopanche* species have been published from southern America to Central America in recent years [35–39]. Our study might provide evidence, based on molecular data, that additional species could potentially be recognized given detailed morphological and field observations as started by Hatt et al. [30]. Indeed, morphologically similar species to *H. abyssinica* have been published in the past, namely *H. sinandevu* (not sampled here) as well as *H. solmsiana* Dinter, and *H. johannis* Becc. The latter two have been treated as synonym of *H. abysssinica* [18, 31]. The first, *H. sinandevu*, is treated as an independent species and parasitizes a different host plant in Kenya (*Commiphora africana* (A. Rich.) Engl., Burseraceae)). In comparison, all other species mentioned above exclusively parasitize Fabaceae [31]. However, further synonyms of *H. abyssinica* exist. According to taxonomic treatments of the last decades, at least eight further synonymous taxa have been described: *Hydnora aethiopica* Decne., *Hydnora angolensis* Decne., *Hydnora abyssinica* A. Braun var. *quinquefida* Engl, *Hydnora bogoscensis* Becc, *Hydnora cornii* Vaccaneo, *Hydnora gigantea* Chiov., *Hydnora hanningtinii* Rendle, and *Hydnora michaelis* Peter [18, 23, 28, 31, 40]. Therefore, it is not unlikely that a greater diversity is currently hidden due to the lack of morphological characteristics and the pooling of taxa based on inaccessible characters.

For the future, an expanded taxon sampling of accessions currently considered *H. abyssinica* and its synonyms, covering the entire known range, is highly desirable to reveal diversity within Hydnora clade II and support further taxonomic revision. Our study showed the viability of molecular data to aide in species deciphering and the present dataset might serve as a starting point to design additional molecular markers to help with species identification in the light of limited morphological data.

### Plastome structural features support phylogenetic findings and systematic revision

Within Hydnoraceae, especially genus *Hydnora*, a fair number of structural plastome differences have been observed, which seem to correlate to specific clades [8]. The proposed Hydnora clade II, comprised of *H. arabica* and Namibian *H. abyssinica* (named *H. hanningtonii* and *H. solmsiana* from now on), is characterized by inverted repeats distinct from the majority of *Hydnora* species, along with two independent translocation and inversion events on the branch leading to it [8]. Phylogenetic inference of the newly sequenced accessions of this study reconstructs their placement within this Hydnora clade II (Fig. 2, Additional file 2: Fig. S1). This placement is fully supported based on bootstrap values as well as supported by structural plastome elements. The sampled *H. abyssinica, H. hanningtonii*, and *H. solmsiana* share two independent events of sequence translocation and inversion (*rps*3 – *rpl*16 region and *rpl*14) compared to the remainder of the Hydnoraceae published to date, along with an inversion of *rps*7 [8]. This adds support to the hypothesis made by Jost et al. [8], proposing that these events happened on the branch leading to this clade and prior to the diversification within. Two major structural differences within the clade can be observed however: (i) the inversion of the *rps*12 exon 2 of *H. solmsiana* (Namibia) compared to *H. hanningtonii* (Oman) and *H. abyssinica* (Kenya), and (ii) the distinct IR gene content for each of the three (Fig. 3).

Our findings indirectly support the new systematic treatment of *Hydnora* [31] as otherwise *H. abyssinica* would render non-monophyletic, as *H. arabica* is nested within. In line with the topological placement and structural plastome evidence is the result of the pairwise distance analysis, which highlights a larger distance of *H. solmsiana* to the remainder of Hydnora clade II than for *H. hanningtonii* to the Kenyan accessions (Additional file 3: Table S2). Based on the data at hand and little variation within the newly sequenced accessions, even on full plastome level (Additional file 3: Table S2), we are however not able to confidently resolve the relationships among the Kenyan accessions further; nuclear markers will potentially be required.

### Hydnoracae plastome evolution

The seven reconstructed plastomes of Kenyan *Hydnora abyssinica* accessions reveal a striking degree of congruence, both on structural and sequence level (Additional file 1: Table S1 and Additional file 3: Table S2). All share the identical quadripartite structure with four genes duplicated within the IRs (*rrn*4.5, *rrn*5, *rrn*23, *rpl*16), and an identical gene order and orientation. With an average length of 24,549 bp, the plastomes of Kenyan *H. abyssinica* place on the lower size range within Hydnoraceae, together with *H. esculenta* (24,479 bp), *H. hanningtonii* (24,790 bp), and *H. solmsiana* (24,672, Jost et al. [8]). The studied accessions contain a set of 24 unique genes (Fig. 1), identical with other published *Hydnora* [7, 8]. Together, Hydnoraceae possess some of the most condensed parasitic plastid genomes known to date [6–8], for example “*Pilostyles* (Apodanthaceae) [3, 4], and *Balanophora* (Balanophoraceae) [5]”.

Adaptation to the holoparasitic lifestyle leads to an extreme bias for Adenosine and Thyrosine nucleotides, compared to the generally already AT rich plastomes of autotrophic plants. This compositional bias affects both coding and non-coding regions [9] as well as all three codon positions, resulting in notable changes in codon usage. Hydnoraceae plastomes consist to ~ 76–80% of AT (Additional file 1: Table S1, [6–8], complemented by an extreme nucleotide bias for chosen synonymous codons (Additional file 4: Table S3). This compositional bias could be the result of the general trend towards A and T nucleotides during a plants adaptation to a heterotrophic lifestyle [9], or be to compensate for limited tRNA import after the plastome-encoded tRNA genes have been lost. In eukaryotes, the codons of highly expressed genes are best recognized by the most abundant tRNAs, resulting from a translation efficiency selection [41, 42]. Codon usage between *Hydnora* and *Prosopanche* is congruent with mostly identical prevalence for each of the synonymous codons per amino acid (Additional file 4: Table S3). Even though the *Prosopanche* plastomes maintain two additional tRNAs (*trn*Y-GUA and *trn*W-CCA) compared to *Hydnora* [6, 8], the codons corresponding to *trn*Y and *trn*W do not show significantly higher usage in *Prosopanche* compared to the sister genus (Additional file 4: Table S3). However, since the RSCU analysis reveals that these codons are clearly being used in *Hydnora*, but lost from their plastome, an import mechanism for them has to exist. To service a specific codon, the corresponding tRNA as binding partner is required or an unspecific one that is able to read multiple codons, following the wobble [43] or superwobble rules [44]. An import for these two tRNAs either could be *Hydnora*-specific or have already existed in a condensed Hydnoraceae MRCA plastome, resulting in non-essential *trn*Y and *trn*W copies in *Prosopanche*. Such a cytosolic import is likely to already exist for most of the other tRNAs lost, yet still used by the Hydnoraceae plastomes. This theory gains additional support by point mutation altering the anticodon of *trn*W in *P. americana*, rendering it putatively non-functional [6].

## Conclusions

We newly sequenced and assembled for the first time plastid genomes of seven *H. abyssinica* accessions from Kenya and compared them with publicly available Hydnoraceae plastomes. Based on multiple lines of evidence and following a taxonomic revision by Hatt et al. [31], we support the recognition of *H. solmsiana* in Namibian (previously recognized as *H. abyssinica*) and *H. hanningtonii* (the correct name of *H. arabica*) in Oman. The plastomes of Kenyan *H. abyssinica* are identical to one another with respect to gene content and quadripartite plastome structure, with little variation on sequence level. Plastome comparison reveals that the orientation of the *rps*12 exon 2 is the only exception to an otherwise congruent gene order and orientation within Hydnora clade II. The exon is inverted in *H. solmsiana* compared to the *H. abyssinica* and *H. hanningtonii*, serving as a structural feature supporting phylogenetic findings. The phylogenetic tree reconstruction based on an 83-plastid loci alignment displayed *H. solmsiana* sister to a clade comprising *H. hanningtonii* and *H. abyssinica*, support the segregation of *H. solmsiana* from the synonymy of *H. abyssinica*, as proposed in the taxonomic revision of the genus. An expanded taxon sampling considering *H. abyssinica* and its relatives, covering the entire distribution range is highly desirable to uncover the diversity within Hydnora clade II and further aid a taxonomic revision. The present dataset might serve as a starting point to design additional molecular markers to help with species identification in the light of limited morphological data. However, a complementary and complete morphological reinvestigation is required as well.

## Materials and methods

### Sample collection and DNA extraction

Seven populations of *H*. *abyssinica* (H0 – H6) were collected by the authors with field assistance by scientists from the National Museum of Kenya at Mt. Kasigau (Taita Hills), a mountain range located in the Taita-Taveta County in south-eastern Kenya. The formal identification of the plant was done by Professor Guang Guang-Wan Hu from Wuhan Botanical Garden, Chinese Academy of Sciences with the help of National Museums of Kenya senior Scientist Dr. Geofrey Mwachala. These locations can be characterized as dry savanna grassland marked by *Fabaceae* trees (e.g. *Albizia* Durazz., *Delonix* Raf., *Kigelia* DC., *Piliostigima* Hochst.), and Euphorbiaceae species (e.g. *Euphorbia tirukali* L.) as well as in the Tsavo East and West National Park. The corresponding collection information and vouchers are provided in Additional file 5: Table S4. Samples were deposited at the Wuhan Botanical Garden herbarium. Within the Taita Hills mountain range, *Hydnora* could not be observed at Ngangao, Iyale, Msidunyi, or Ndiwenyi, but exclusively at Mt. Kasigau, Voi, Mbolo/Ronge juu, Kishushe, and Ndii near the border of Kenya and Tanzania. Collection permits were obtained under the Sino Africa Joint Research Center in 2019. Genomic DNA was isolated using a cetyltrimethylammonium bromide method [45]. All sampled Kenyan *Hydnora abyssinica* were growing on *Vachelia*, *Albizia*, and *Kigelia*.

### Illumina sequencing and plastome assembly

The *H*. *abyssinica* libraries were constructed with an insert size of 250 bp, following the manufacturer’s standard method of TruSeq DNA sample preparation kits and was sequenced on an Illumina Hiseq-2000 platform at Novogene Co (Beijing, China). Low-quality data and adaptors were filtered and trimmed (quality scores < 20 were trimmed). We obtained between ~ 45 mio. raw reads for accession *H. abyssinica* H0 and ~ 22 mio reads for accessions H1-H6.

The sequence raw data were assembled using both a *de novo* assembly and a seed-based assembly approach. The *de novo* assemblies were done using CLC Genomics workbench (v. 11.1, Biomatters, Ltd., New Zealand), allowing for automatic estimation of optimal bubble and word sizes for each sample. Subsequently, to verify the correctness of the assemblies, readmappings were conducted using the same software. The assemblies were filtered for contigs with putative plastid origin (BLASTn, E-value 1e-10). Seed-based reconstructions of plastid scaffolds were carried out in GetOrganelle (v. 1.7.4.1, [46] using standard settings. The filtered contigs as well as the plastome scaffolds created by GetOrganelle were imported into Geneious (v. 11.1.5, Biomatters, Ltd., New Zealand), where they were further assembled into complete plastid genomes.

### Primer design and plastome verification

To verify plastome regions with low read coverage, to bridge assembly gaps, and to verify IR boundaries we designed primers (Additional file 5: Table S5) using PhyDE (Phylogenetic Data Editor, [47]). The Polymerase chain reaction (PCR) amplification was carried out on a A300 thermal cycler (Hangzhou Long Gene Scientific Instruments Co., Ltd.) with the following settings: denaturation at 98ºC for 2 min, followed by 35 cycles of 98ºC for 10 s, 10 s annealing at 57ºC, 45 s extension at 72ºC, and a final extension step at 72ºC for 5 min. The amplifications were performed in 40 µL reactions, containing 20 µL 2xT8 High-Fidelity Master Mix (Tsingke Biotechnology Co., Ltd.), 2 µL of each primer, 1 µL DNA template, and 15 µL double distilled water. A DNA agarose gel extraction kit was used to clean the PCR products after separation by electrophoresis on a 0.5% agarose gel. Products were then Sanger sequenced as both the forward and reverse strand on an ABI 3730 XL capillary electrophoresis sequencer (Tsingke Biotechnology Co., Ltd., Wuhan, China). The sequenced products were then aligned to the respective plastome regions after manually checking the respective chromatogram.

### Genome annotation and visualization

Newly sequenced and assembled plastomes were annotated in Geneious (v. 11.1.5, Biomatters, Ltd., New Zealand), using the published plastome of *H. abyssinica* (NC_065049) [8]. Annotations were manually inspected and adjusted where necessary. For protein-coding genes, annotations contain complete open reading frames, including a start and stop codon. The plastomes were visualized as both circular and linear structures using OGDRAW (Organellar Genome DRAW)[48].

### Phylogenetic analyses

To reconstruct the phylogenetic placement of the seven newly sequenced *H. abyssinica* accessions, we created alignments of 83 protein-coding and rRNA genes based on the data set of [8], including a representative sampling among ANA-grade taxa, magnoliids, eudicots, and monocots. Hydnoraceae are represented with 21 genes. Alignments were constructed using the MAFFT algorithm (v. 7.450, [49, 50] and manually adjusted using AliView (v. 1.20) [51]. All genes were subsequently concatenated using Geneious (v. 11.1.5, Biomatters, Ltd., New Zealand) and inferences were calculated using RAxML (v. 8.2.12) [52] implemented in Cipres Science Gate [53, 54], applying a gene partitioning approach. The best-fitting nucleotide substitution model was estimated prior using PartitionFinder2 [55, 56]. Tree files were visualized using TreeGraph 2 [57].

### Relative synonymous codon usage

The relative synonymous codon usage (RSCU, [58]) was estimated for the set of Hydnoraceae protein coding genes using the Sequence Manipulation Suite (SMS,[59]). The comparison was done among the Hydnoraceae sampling of Jost et al. [8] and the newly sequenced *H. abyssinica* H0 of this study as well as closely related heterotrophic taxa (*Aristolchia fimbriata* Cham. CM034085, *Lactoris fernandeziana* Phil. NC_065383, and *Thottea sumatrana* (Merr.) Ding Hou NC_065017). Accession *H. abyssinica* H0 serves as a representative for the six other, newly sequenced accessions. The RSCU is estimated as the observed frequency of a specific codon divided by its expected frequency, assuming equal codon usage for synonymous codons.

## Electronic supplementary material

Below is the link to the electronic supplementary material.

Supplementary Material 1

Supplementary Material 2

Supplementary Material 3

Supplementary Material 4

Supplementary Material 5

Supplementary Material 6

## Data Availability

All the accession numbers and the newly sequenced (OP495727,OP495728,OP495729,OP495730,OP495731,OP495732,OP495733) and assembled plastomes for seven accessions of *Hydnora abyssinica* have been uploaded to the National Center for Biotechnology Information database as attached to the additional file Table S5.

## Abbreviations

BI: Bayesian Inference
BS: Bootstrap support
CTAB: Cetyltrimethylammonium bromide
IR (IRa/IRb): Inverted repeats
LSC: Large single copy
ML: Maximum Likelihood
PCR: Polymerase chain reaction
RSCU: Relative synonymous codon usage
rRNA: Ribosomal RNA genes
SAJOREC: Sino Africa Joint Research Center
SSC: Short small single copy
tRNA: Transfer RNAs genes
HIB: Wuhan Botanical Garden
