# Supplementary figures and images for: Phylogenetic and comparative analyses of Hydnora abyssinica plastomes provide evidence for hidden diversity within Hydnoraceae
Source: BMC Ecol Evol. 2023 Jul 18;23:34. doi: 10.1186/s12862-023-02142-w (PMC10353213; doi:10.1186/s12862-023-02142-w)

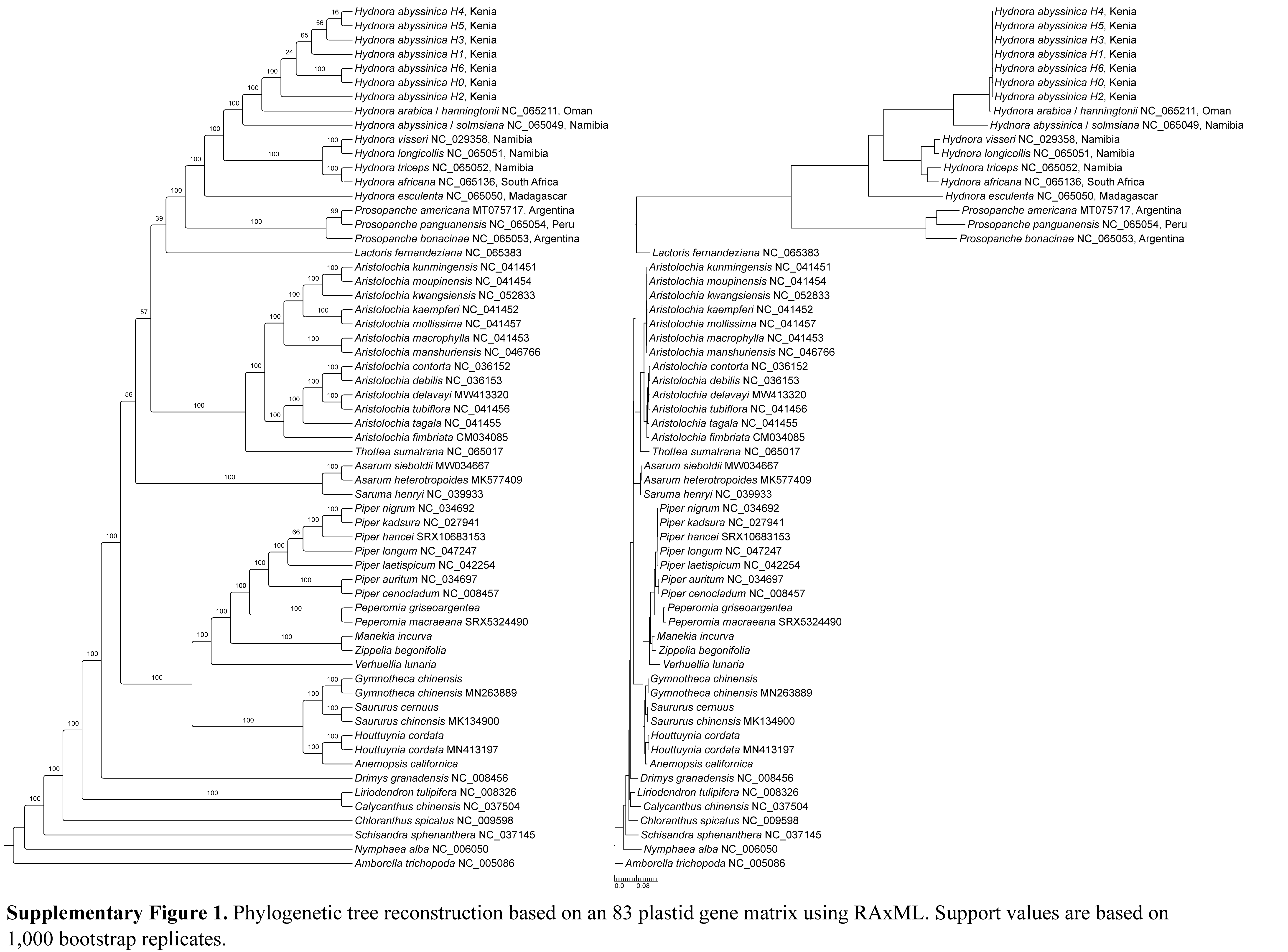

Supplement: Supplementary file 4 — Supplementary Material 4 [file 12862_2023_2142_MOESM4_ESM.png]
