# Supplementary material for: Phylogenetic and comparative analyses of Hydnora abyssinica plastomes provide evidence for hidden diversity within Hydnoraceae
Source: BMC Ecol Evol. 2023 Jul 18;23:34. doi: 10.1186/s12862-023-02142-w (PMC10353213; doi:10.1186/s12862-023-02142-w)
Supplement: Supplementary file 6 — Supplementary Material 6 [file 12862_2023_2142_MOESM6_ESM.docx]

**Table S4.** The collection information corresponding to the newly sequenced accessions. The accession number labeling corresponds to MK – first two initials of the last name of the collector, SAJOREC –the research project title acronym, SAJIT – stands for the Sino-African team, and last is the running number individually assigned by the respective collector. Herbarium acronym follows Index Herbarium: National Museums of Kenya (EA) and Wuhan Institute of Botany (HIB).

| **Species** | **Lab**  **number** | **GPS coordinates** | | **Kenyan origin** | **Accession number (herbarium acronym)** |
| --- | --- | --- | --- | --- | --- |
|  |  | **Longitude** | **Latitude** |  |  |
| *Hydnora abyssinica* | H0 | 38.652904 | -3.784301 | Kasigau region (Taita-Taveta county) | MKSAJOREC/SAJIT-H0 (EA, HIB) |
| *H. abyssinca* | H1 | 38.55609 | -3.39625 | Kasigau region (Taita-Taveta county) | MKSAJOREC/SAJIT-001 (EA, HIB) |
| *H. abyssinca* | H2 | 38.752904 | -3.803301 | Kasigau region (Taita-Taveta county) | MKSAJOREC/SAJIT-002 (EA, HIB) |
| *H. abyssinca* | H3 | 38.644429 | -3.786453 | Kasigau region (Taita-Taveta county) | MKSAJOREC/SAJIT-003 (EA, HIB) |
| *H. abyssinca* | H4 | 38.656427 | -3.791164 | Kasigau region (Taita-Taveta county) | MKSAJOREC/SAJIT-004 (EA, HIB) |
| *H. abyssinca* | H5 | 38.633546 | -3.80741 | Kasigau region (Taita-Taveta county) | MKSAJOREC/SAJIT-005 (EA, HIB) |
| *H. abyssinca* | H6 | 38.64638 | -3.792654 | Kasigau region (Taita-Taveta county) | MKSAJOREC/SAJIT-006 (EA, HIB) |
